# The effect of type 2 diabetes mellitus on the prognosis of osteoporotic vertebral compression fracture with osteoporotic fracture classification after vertebroplasty

**DOI:** 10.1186/s13018-023-03792-8

**Published:** 2023-05-09

**Authors:** Zixiang Wang, Hanquan Wang, Chenyang Zhuang, Weisin Chen, Tien-Manh Hoang, Juan Li, Hong Lin

**Affiliations:** 1 Department of Orthopedics, Zhongshan Hospital, Fudan University, Shanghai, 200032 China; 2 Department of Orthopedics, Shanghai Geriatrics Medical Center, Fudan University, Shanghai, 201100 China

**Keywords:** Osteoporotic vertebral compression fracture, Type 2 diabetes mellitus, Osteoporotic fracture classification, Percutaneous vertebroplasty, Prognosis

## Abstract

**Background:**

To analyze the clinical and radiological effects of type 2 diabetes mellitus on the prognosis of osteoporotic vertebral compression fracture after percutaneous vertebroplasty, and explore the prognostic value of osteoporotic fracture classification.

**Methods:**

Osteoporotic vertebral compression fracture patients who received vertebroplasty from January 1, 2016 to June 30, 2021 were divided into type 2 diabetes mellitus group and control group in this retrospective cohort study. Visual analogue scale, Oswestry Disability Index, bone cement leakage, new compression fracture, anterior, middle, and posterior portion heights of vertebral body and local Cobb angle on X-ray before surgery, 2 days after surgery, 6 months, and 12 months after surgery were recorded, and the osteoporotic fracture classification was performed.* P* < 0.05 was set as statistical significance.

**Results:**

A total of 261 vertebral bodies were included, containing 68 in the type 2 diabetes mellitus group and 193 in the control group. There were no differences in baseline characteristics between the two groups. At 6 months after vertebroplasty, the local Cobb angle of the type 2 diabetes mellitus group was 8.29 ± 4.90° greater than that of the control group 6.05 ± 5.18° (*P* = 0.002). At 12 months, compared with pre-operation, the anterior portion height recovered 8.13 ± 12.90%, which was less than 12.51 ± 14.92% of the control group (*P* = 0.032), and 19.07 ± 16.47% of the middle portion height recovery was less than the control group’s 24.63 ± 17.67% (*P* = 0.024). Compared with the control group, osteoporotic fracture 2 vertebral bodies of the type 2 diabetes mellitus group at 12 months postoperatively in middle portion height (14.82 ± 14.71% vs 24.78 ± 18.16%, *P* = 0.023) and local Cobb angle (5.65 ± 4.06° vs 3.26 ± 4.86°,* P* = 0.043) restored significantly worse. Besides, osteoporotic fracture 3 with type 2 diabetes mellitus restored worse in anterior portion height (5.40 ± 11.02% vs 13.57 ± 12.79%, *P* = 0.008), middle portion height (11.22 ± 15.53% vs 17.84 ± 12.36%, *P* = 0.041) and local Cobb angle (10.85 ± 3.79 vs 7.97 ± 3.83°, *P* = 0.002) at 12 months postoperatively. There was no difference in radiological outcomes of osteoporotic fracture 4 between the two groups.

**Conclusions:**

The degree of fractured vertebral compression, the recovery of the height and angle obtained immediately after surgery and the clinical symptoms in type 2 diabetes mellitus patients were not different from those in the control. However, vertebral body morphology of type 2 diabetes mellitus patients was worse since the sixth month after surgery. Osteoporotic fracture classification has a good prognostic reference value for both the control and the type 2 diabetes mellitus population.

**Supplementary Information:**

The online version contains supplementary material available at 10.1186/s13018-023-03792-8.

## Introduction

Osteoporosis (OP) is a common systemic and metabolic disease characterized by decreased bone mass, altered bone microarchitecture, and weakened biomechanical properties. With the intensification of the aging phenomenon in China, the prevalence of OP has gradually increased, reaching 32.0% of the population over the age of 65, ranking first in the world [1]. The prevalence of various complications has also increased. Osteoporotic fractures caused by mild external forces are most common, mainly occurring in the spine and hip, and the number of new cases in the United States exceeds 1.5 million each year [2]. Osteoporotic vertebral compression fracture (OVCF) often occurs in the thoracic and lumbar spine, which can cause severe back pain, muscle spasm and atrophy, kyphosis, and respiratory dysfunction. The mortality rate of women over 65 years old with OVCF is 23% higher than that of the control group, and it increases with the number of fractured vertebrae [3]. Percutaneous vertebroplasty (PVP) has been one of the common treatments for OVCF in the past decades, which provides rapid pain relief through minimally invasive methods. And it could stabilize fractures, restore mechanical strength of the vertebral body, prevent further compression and help patients recommence normal activities.

Type 2 diabetes mellitus (T2DM) accounts for more than 90% among all diabetes patients. According to the estimates by the International Diabetes Federation, the total number of people with diabetes between the ages of 20 and 79 in the world has reached 426 million in 2019, of which China, the country with the largest number, accounts for 140 million [4]. With the function of pancreatic β-cells progressively damaged, chronic hyperglycemia and insulin resistance could cause polydipsia, polyuria and polyphagia. Metabolic disorders in long-term result in different complications of various organs such as kidneys, bones, feet, eyes, and heart. Previous experiments have shown that T2DM can promote the progression of OP by disrupting the balance of osteogenesis, forming an inflammatory and reactive oxygen species microenvironment, interfering with the metabolism of minerals such as calcium and phosphorus, and inhibiting bone microvascular blood supply [5, 6]. Clinical studies by Hofbauer et al. proved that T2DM and osteoporotic fractures have a synergistic effect. Patients with OP and T2DM have a significantly increased risk of falling. And the risk of fracture is 4 times that of the normal population, and the risk of OVCF is 1.35 times that of the normal population [7]. Chen further demonstrated that T2DM is a risk factor for adjacent vertebral fractures in OVCF patients after vertebroplasty [8]. In addition, high blood glucose may lead to infection, wound complications, etc. However, there is no previous study to evaluate the prognosis of clinical symptoms and vertebral body morphology in patients with OVCF and T2DM after PVP.

**Fig. 1:**
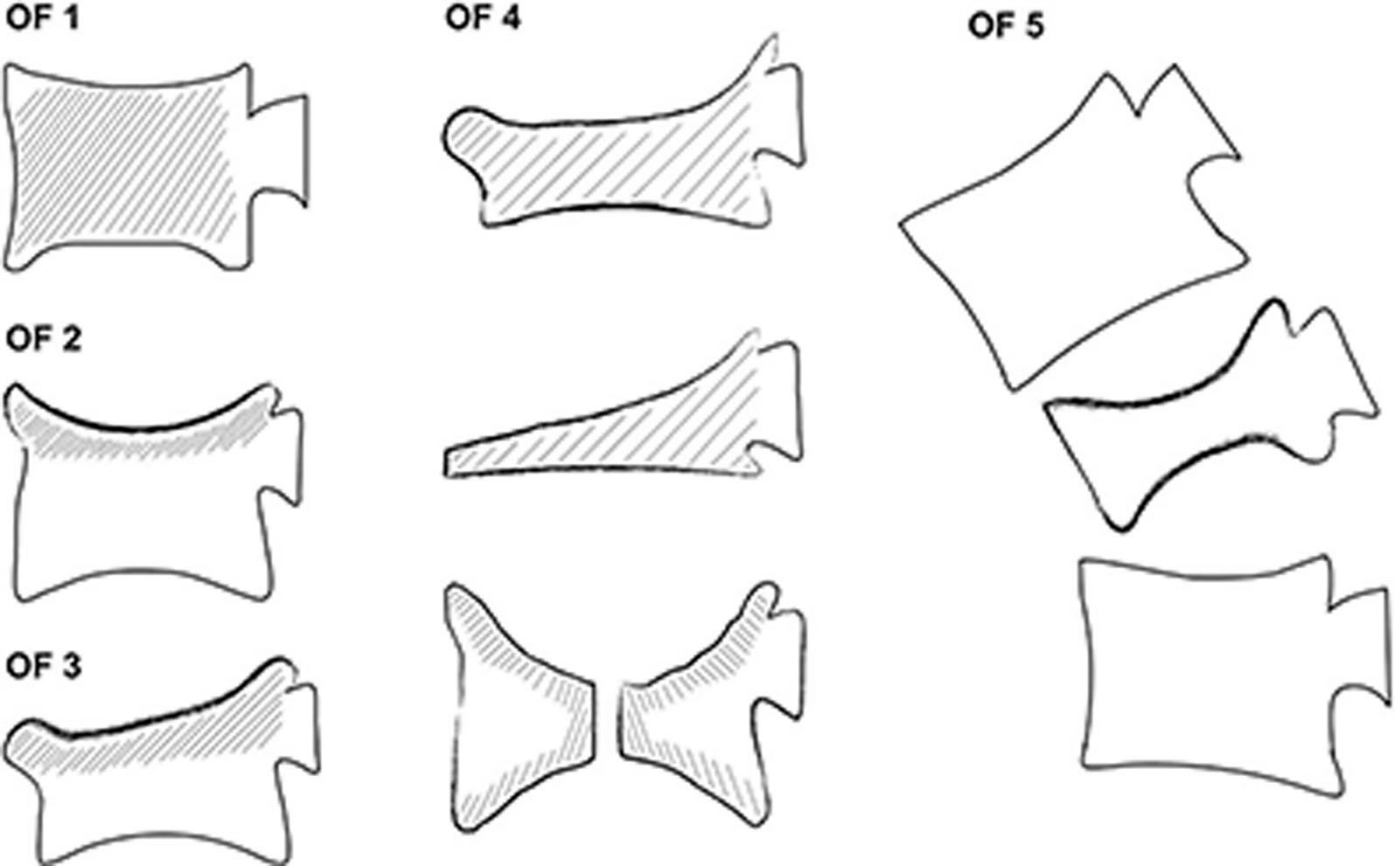
Schematic diagram of vertebral body morphology of the OF classification [9]. OF, osteoporotic fracture

The osteoporotic fracture (OF) classification of OVCF is a relatively new preoperative grading method, which was published in 2018 by the German Society for Orthopedics and Trauma for the first time [9]. It classifies fractured vertebral bodies into grades 1–5 according to biomechanical stability and morphological changes (Fig. 1), which is easier to utilize and easier to distinguish morphologies than previous grading systems [9]. Although the authors have given recommendations of treatment for all levels in the literature, the prognostic value of OF classification is still unclear, and its clinical value is limited.

Therefore, this original research retrospectively analyzed the prognostic effects of T2DM on the clinic and radiology of OVCF patients after PVP, focusing on changes in the height and angle of fractured vertebrae, subjective pain assessment and complications. We aim to indicate the efficacy of PVP surgery in diabetic patients and to preliminarily study the prognostic significance of OF classification, which would help clinicians to provide standardized and comprehensive therapeutic schedules for patients.

## Materials and methods

This retrospective cohort study included baseline data, laboratory and imaging data, and was approved by the institutional review board and independent ethics committee of Zhongshan Hospital, Fudan University. All eligible participants in the study gave written informed consent.

### Patients

We included patients with OVCF who received PVP in the Department of Orthopedics of our hospital from January 1, 2016 to June 30, 2021. The enrolled patients were included in the T2DM group according to their past T2DM history or two fasting plasma glucose ≥ 7.0 mmol/L on different days, and the rest of the patients were included in the control group.

The inclusion criteria were as follows: (1) PVP for acute thoracic or/and lumbar OVCF caused by minor trauma or falling from height not above normal standing; (2) diagnosis of OP by dual energy X-ray absorptiometry (DXA) with *T*-score of − 2.5 or lower at lumbar spine, femoral neck, or total hip, or previous history of OVCF, proximal femoral fracture, or other fragility fracture [10]; (3) 50 years old ≤ age ≤ 90 years old.

The exclusion criteria were as follows: (1) trauma caused by severe violence, such as car accident, fall from high place, etc.; (2) other non-OVCF fractures; (3) long-term or recent use of heparin, anti-OP drugs, glucocorticoids or other drugs that may affect bone metabolism; (4) chronic liver/renal insufficiency or malignant tumors and other diseases that may affect bone metabolism; (5) have received other spinal surgery within 6 months; (6) preoperative baseline measurements suggest abnormal blood calcium level: lower than 2.00 mmol/L or greater than 2.75 mmol/L, or abnormal 25-hydroxy vitamin D level: < 15 ng/mL; (7) Because previous studies have shown that the imaging results of chronic OVCF are significantly worse than the acute, patients with no apparent onset of symptoms within 6 weeks prior to surgery were excluded to reduce bias [11, 12].

### Surgery and post-operative care

All eligible patients were operated on by the same surgical team and the same chief surgeon. PVP procedures for the treatment of OVCF lesions were performed according to the standard surgical procedure in previous literature [13]. The full set of medical equipment was provided by Huakerun Biotechnology Co., Ltd., Ningbo, China.

The fractured vertebra was located by C-arm X-ray imaging. The front and lateral fluoroscopic monitoring of the compressed vertebral body is performed during the penetration, to ensure that the puncture needle is always in the pedicle and to avoid penetrating out of the vertebral body. When the needle enters the middle and posterior third of the vertebral body, the cannula is inserted in a limited direction to make the distal end to the opposite side of the vertebral body. The amount and injection speed of bone cement polymethyl methacrylate (PMMA) accord to the site and the extent of the lesion. Under the monitoring of C-arm, we stop the injection when PMMA reaches the posterior wall of the vertebral body, to ensure that there’s no leakage to the spinal canal or anterior gap. If the bone cement enters the spinal canal, intervertebral disc or intervertebral foramen during the injection, stop the operation immediately.

Routine postoperative management included dressing change on the first postoperative day, and anteroposterior and lateral spine X-ray examinations on the second day, the sixth month, and the twelfth month after surgery. No oral or intravenous analgesics were routinely administered. Heavy physical labor such as lifting was prohibited for 6 months after surgery. In addition, throughout the follow-up, all participants received oral calcitriol 0.25 μg/d and calcium 1000 mg/d as the basic treatment for OP, and patients were encouraged to get more sunshine exposure and increase outdoor sports. According to the blood examination results of bone metabolism and economic conditions of the enrolled patients, different anti-OP drugs were administered after surgery, such as teriparatide (subcutaneous injection, 20 μg/day, once a day, Eli Lilly, USA) was administered for at least 6 consecutive months; or zoledronic acid aqueous solution (intravenous infusion, 5 mg/time, once a year, Novartis, USA) was applied on the third day after surgery for at least 15 min; or denosumab (subcutaneous injection, 60 μg/time, once every 6 months, Amgen, USA) was administered.

### Clinical data

Baseline characteristics including gender, age, height, weight, body mass index (BMI), chronic diseases such as diabetes or hypertension, mean perioperative fasting blood glucose, preoperative glycosylated hemoglobin (HbA1c), operative segment and segment amount were recorded. Intraoperative information, such as operative time, intraoperative blood loss and bone cement injection volume was also recorded. Oral antidiabetic drug regimens or insulin regimens were recorded in patients with T2DM. The visual analogue scale (VAS) ranges from 0 to 10 for grading low back pain, with higher scores representing more severe pain. The Oswestry disability index (ODI) ranges from 0 to 50, used to assess the neurological function and quality of life. These two clinical parameters were assessed before PVP and at 2 days, 6 months, and 12 months after PVP. Complications such as nerve root injury, spinal cord injury, allergy, spinal infection and pulmonary embolism were observed and recorded if happened.

### Radiological evaluation

**Fig. 2:**
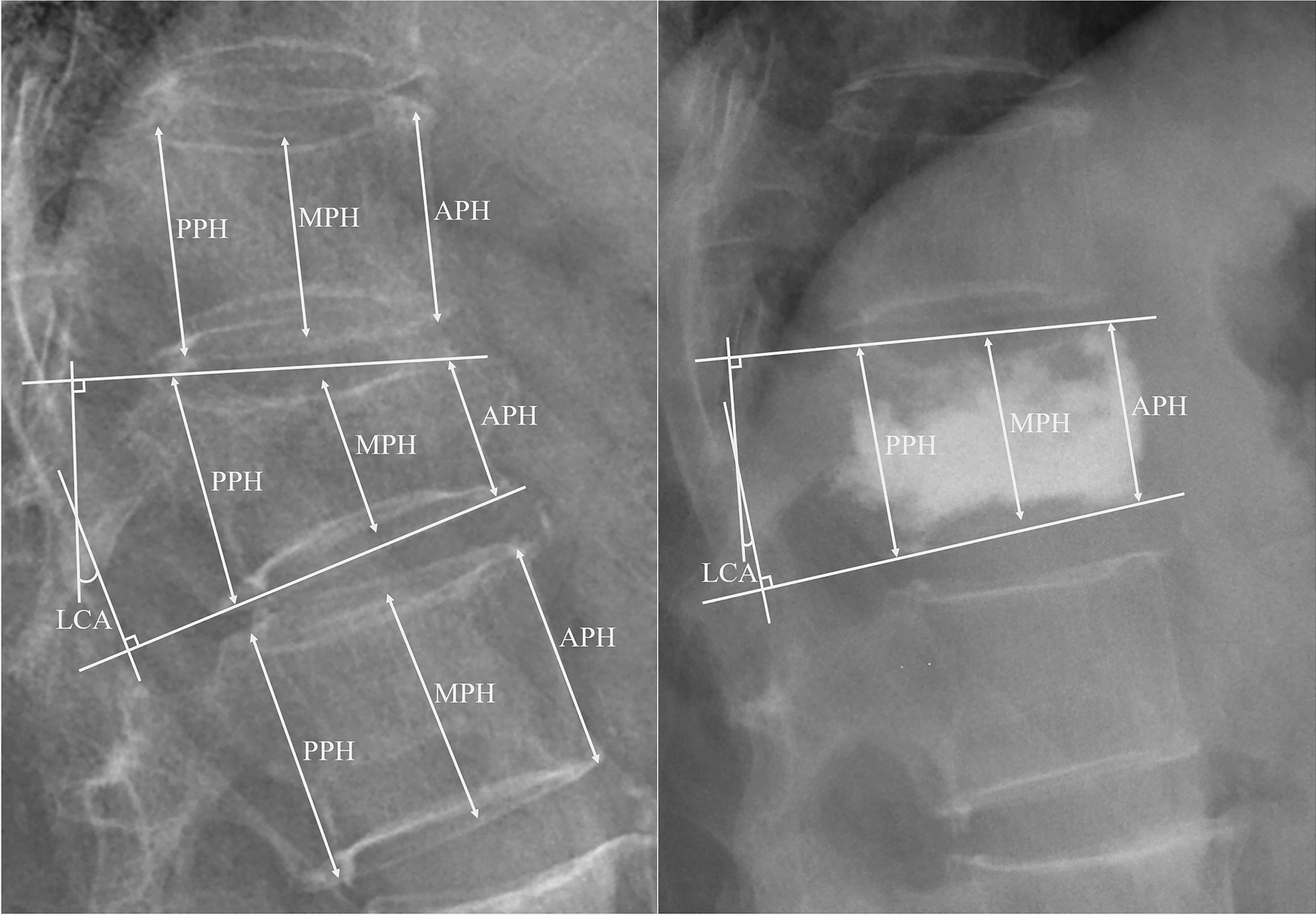
Example of radiological measurements of vertebral body before and after PVP. APH, anterior portion height; LCA, local Cobb angle; MPH, middle portion height; PPH, posterior portion height

Based on lateral vertebral X-rays, two spine surgeons unrelated to the operation independently assessed the imaging parameters of each enrolled patient before PVP and 2 days, 6 months, and 12 months after PVP. The data includes the anterior, middle and posterior portion heights (APH, MPH and PPH) of the fractured vertebral body and its upper and lower unfractured adjacent vertebrae, as well as the local Cobb angle (LCA) of the fractured vertebral body. To minimize the risk of bias, statistical analyses were performed using the mean measurements from the two spine surgeons. The original relative height of each portion of the fractured vertebral body was calculated as the average height of each portion of the nearest upper and lower non-fractured vertebral bodies. Then, the height difference of each portion and local Cobb angle of the fractured vertebral body before and after the operation were calculated. The lost percentage of height is calculated as: height loss (%) = (original height-preoperative height)/original height * 100%, and the percentage of height recovery is calculated as: height recovery (%) = (postoperative height-preoperative height)/original height * 100% to evaluate the efficacy of percutaneous vertebroplasty on OVCF and to compare the difference in prognosis between the T2DM group and the control group. An example of the height of each portion and angle measurement is shown in Fig. 2. Bone mineral density (BMD) was assessed by DXA (Hologic Discovery A device, Bedford, USA) preoperatively or on the second postoperative day. Due to the interference of bone cement in some patients, the mean BMD of the unoperated lumbar vertebrae or hip joints was used to evaluate the bone quality of patients after surgery. During the whole postoperative period, complications such as bone cement leakage, refracture of other vertebral bodies and corresponding imaging examinations were observed and analyzed if happened.

### Statistical analysis

All statistical analyses were performed on the SPSS software (22.0, IBM corp., Armonk, USA). All continuous variable results were displayed in the form of mean ± standard deviation. Paired *t*-test was used to compare the changes of clinical and imaging indexes in each group before and after treatment. Continuous variables such as baseline characteristics, BMD, VAS, ODI, vertebral height and local Cobb angle were compared between the two groups by independent *t*-test, along with the subgroup analysis OF classification. One-way ANOVA and post hoc tests were used to compare the prognostic value of OF classification. Pearson's Chi-square test and Fisher's exact test were used to compare categorical variables such as gender, fracture part, and complications between the two groups. The rank sum test was used to compare multiple ordinal categorical variables between the two groups, such as the number of cases with different amounts of fracture segments, the number of vertebral bodies with different OF grades, etc. Based on two-sided 95% confidence intervals, *P* < 0.05 was set as statistical significance.

## Results

### Baseline characteristics of the enrolled population

**Fig. 3:**
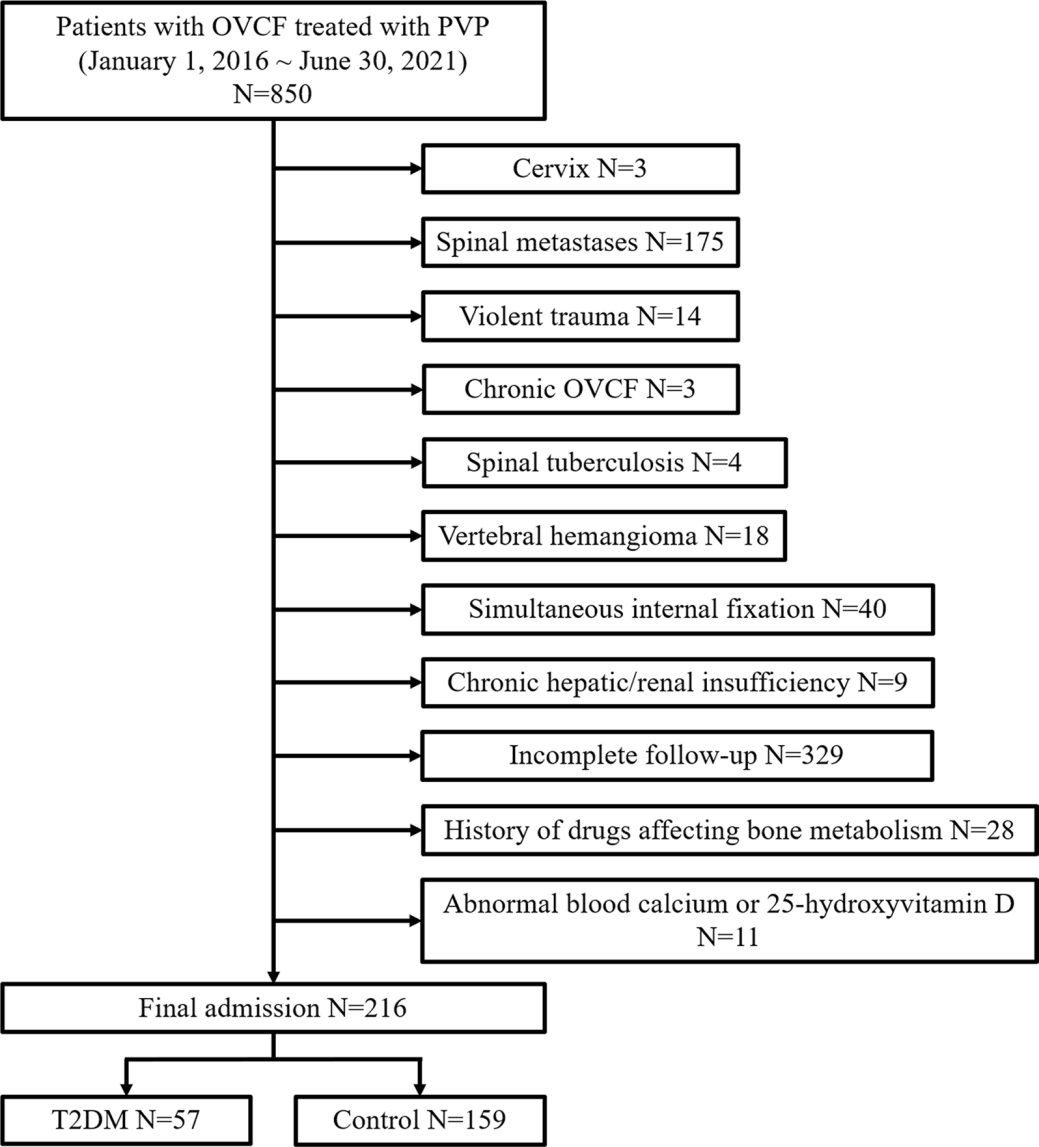
Flow chart of the enrolled patients screening. OVCF, osteoporotic vertebral compression fracture; PVP, percutaneous vertebroplasty; T2DM, type 2 diabetes mellitus

**Fig. 4:**
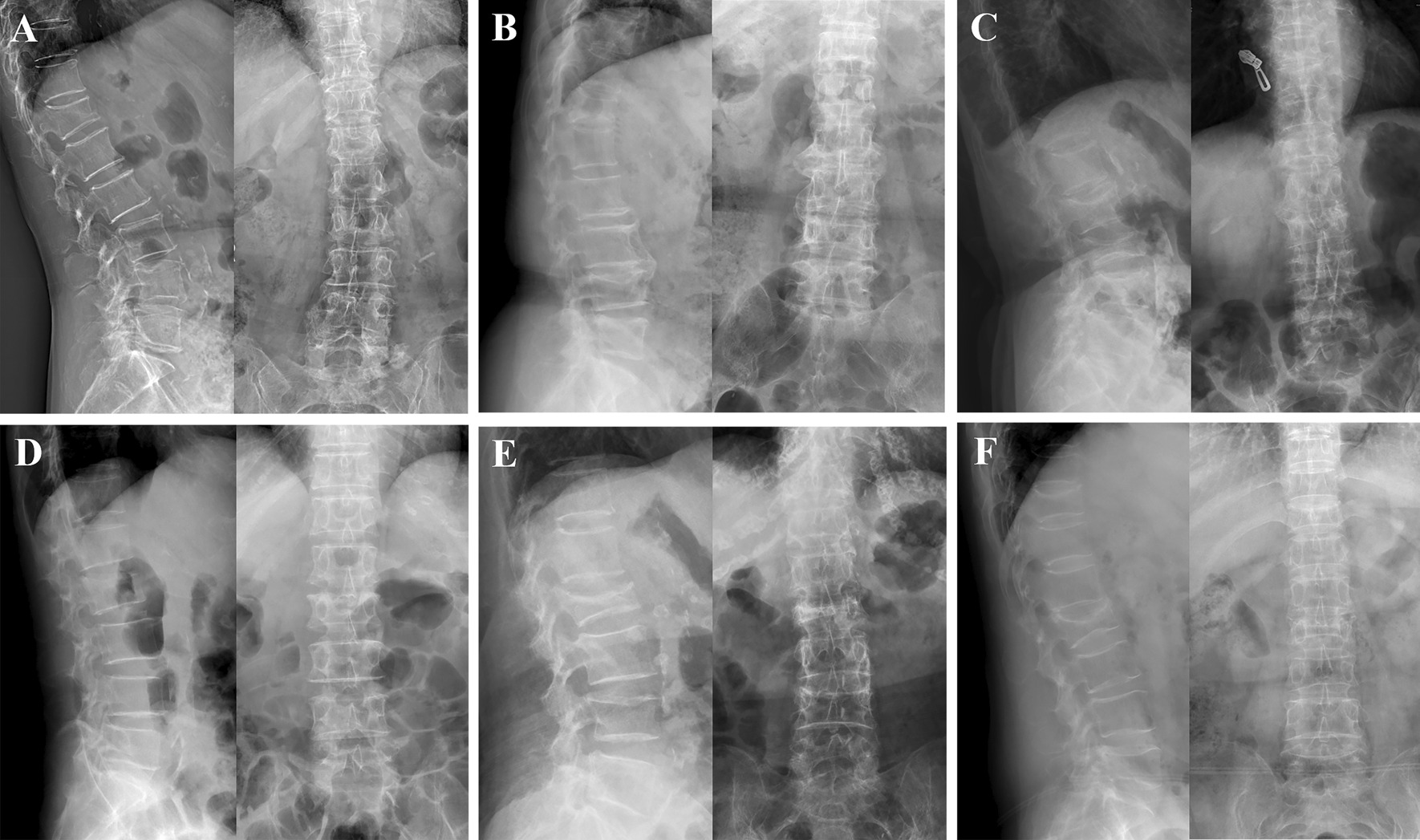
Lateral and anteroposterior vertebral body X-rays of typical cases with different amount of fractured segments in the T2DM group and control group. **A**–**C** for the T2DM cases. **D**–**F** for the control cases. Single segment of T12 fracture in (**A**) and L1 fracture in (**D**). Double segments of T12 and L4 fractures in (**B**) and L2 and L4 fracture in (**E**). Multi-segments (≥ 3) of T12, L2 and L3 fractures in (**C**) and T11, L2 and L5 fractures in (**F**). T2DM, type 2 diabetes mellitus

According to the inclusion and exclusion criteria, of 850 patients who underwent PVP surgery between January 1, 2016 and June 30, 2021, a total of 216 patients with OVCF were eventually included in this study for further analysis (Fig. 3). Table 1 demonstrates the demographic characteristics, surgical information, and fracture segments of the enrolled patients, which included 57 patients with type 2 diabetes (T2DM group) and 159 control patients without diabetes (control group). Gender, age, height, weight, BMI, *T*-value of perioperative BMD, the prevalence of hypertension, the prevalence of coronary heart disease, operation time, intraoperative blood loss, and amount of bone cement injected into each segment showed no statistical significance between the two groups (*P* > 0.05). During hospitalization, the fasting plasma glucose and HbA1c in the T2DM group were 8.48 ± 2.12 mmol/L and 8.16 ± 1.75%, respectively, while the blood glucose and HbA1c in the control group were 5.49 ± 0.99 mmol/L and 5.64 ± 1.03%, respectively. The differences between the two groups were significant (*P* < 0.001). A total of 261 compression fracture segments underwent PVP treatment in this study, including 48 cases of single-segment fractures, 7 cases of double-segment fractures, and 2 cases of three-segment fractures in the T2DM group, with 4 thoracic vertebrae (T5–T10), 48 thoracolumbar vertebrae (T11–L2), and 16 lumbar vertebrae (L3–L5). In the control group, there were 130 single-segment fractures, 25 double-segment fractures, 3 three-segment fractures, and 1 four-segment fracture, with 15 thoracic vertebrae, 127 thoracolumbar vertebrae, and 51 lumbar vertebrae. There was no significant difference between the two groups. The lateral and anteroposterior X-ray film of the typical cases with the different amount of fractured segments of the two groups are shown in Fig. 4. The information of the detailed segments of every included de-identified participant was described in the Additional file 1. Moreover, no adverse events or serious adverse events, such as palpitations, nausea, vomiting, myalgia, malaise, and death, were observed or recorded after operation.

**Table 1:** Baseline characteristics of patients in the T2DM group and the control group

	T2DM	Control	*P* value
Age (years)	73.19 ± 8.18	72.89 ± 9.51	0.833
Gender (Male/female)	16/41	31/128	0.178
Height (cm)	162.79 ± 8.76	160.96 ± 7.90	0.146
Weight (kg)	61.71 ± 11.36	59.76 ± 10.71	0.248
BMI (kg/m^2^)	23.31 ± 3.32	23.02 ± 3.52	0.711
Lumbar BMD (*T* value)	− 3.59 ± 1.02	− 3.84 ± 0.95	0.444
Hip BMD (*T* value)	− 3.38 ± 0.57	− 3.59 ± 0.92	0.377
Blood glucose (mmol/L)	8.48 ± 2.12	5.49 ± 0.99	< 0.001*
HbA1c (%)	8.16 ± 1.75	5.64 ± 1.03	< 0.001*
Hypertension (N)	27	62	0.270
Coronary heart disease (N)	6	8	0.148
Operation time (min)	29.67 ± 8.47	28.42 ± 6.58	0.260
Intraoperative blood loss (ml)	8.42 ± 4.92	8.08 ± 4.78	0.643
Bone cement volume (ml/segment)	5.70 ± 1.90	5.45 ± 1.85	0.338
Fractured segments (*N*)	68	193	0.801
Thorax (T5–T10)	4	15	
Thoracolumbar (T11–L2)	48	127	
Lumbar (L3–L5)	16	51	
Fractured cases (*N*)	57	159	0.709
Single segment	48	130	
Double segments	7	25	
Multi-segments (≥ 3)	2	4	

*BMD*, bone mineral density; *BMI*, body mass index; *HbA1c*, glycosylated hemoglobin; *T2DM*, type 2 diabetes mellitus

*P < 0.05

### Clinical outcomes

A total of 209 (96.76%) among the 216 patients returned to their activity level before fracture on postoperative day 2, and 100% returned to full activity on postoperative day 5. On the second day after PVP, the mean VAS of patients in the T2DM group decreased from 7.54 ± 1.61 to 3.01 ± 1.36, and the mean ODI decreased from 38.09 ± 4.77 to 21.39 ± 2.73. The mean VAS of the control group decreased from 7.62 ± 1.42 to 3.05 ± 1.27, and the mean ODI decreased from 37.39 ± 4.22 to 21.81 ± 3.10. Therefore, the clinical symptom scores of low back pain in both groups were significantly lower than those before operation (*P* < 0.001), and there were statistical differences between 6 and 12 months after PVP (*P* < 0.001). The clinical symptom scores of the two groups at 6 and 12 months after surgery were also significantly lower than those on the second day after surgery (*P* < 0.001). Besides, there were no statistically significant differences in VAS and ODI before PVP or at any three postoperative time points between groups (Table 2).

**Table 2:** Clinical parameters of patients in the T2DM group and control group before surgery, 2 days, 6 months and 12 months after surgery

	Before	2 days	6 months	12 months
*VAS*				
T2DM	7.54 ± 1.61	3.01 ± 1.36	1.81 ± 1.03	1.7 ± 0.82
Control	7.62 ± 1.42	3.05 ± 1.27	1.65 ± 0.97	1.67 ± 0.96
*P* value	0.734	0.850	0.296	0.840
*ODI*				
T2DM	38.09 ± 4.77	21.39 ± 2.73	10.42 ± 1.38	10.93 ± 1.51
Control	37.39 ± 4.22	21.81 ± 3.10	10.25 ± 1.64	10.55 ± 1.77
*P* value	0.302	0.361	0.486	0.154

*ODI*, Oswestry disability index; *T2DM*, type 2 diabetes mellitus; *VAS*, visual analogue scale

### Comparison of radiological results of T2DM group and control group

**Fig. 5:**
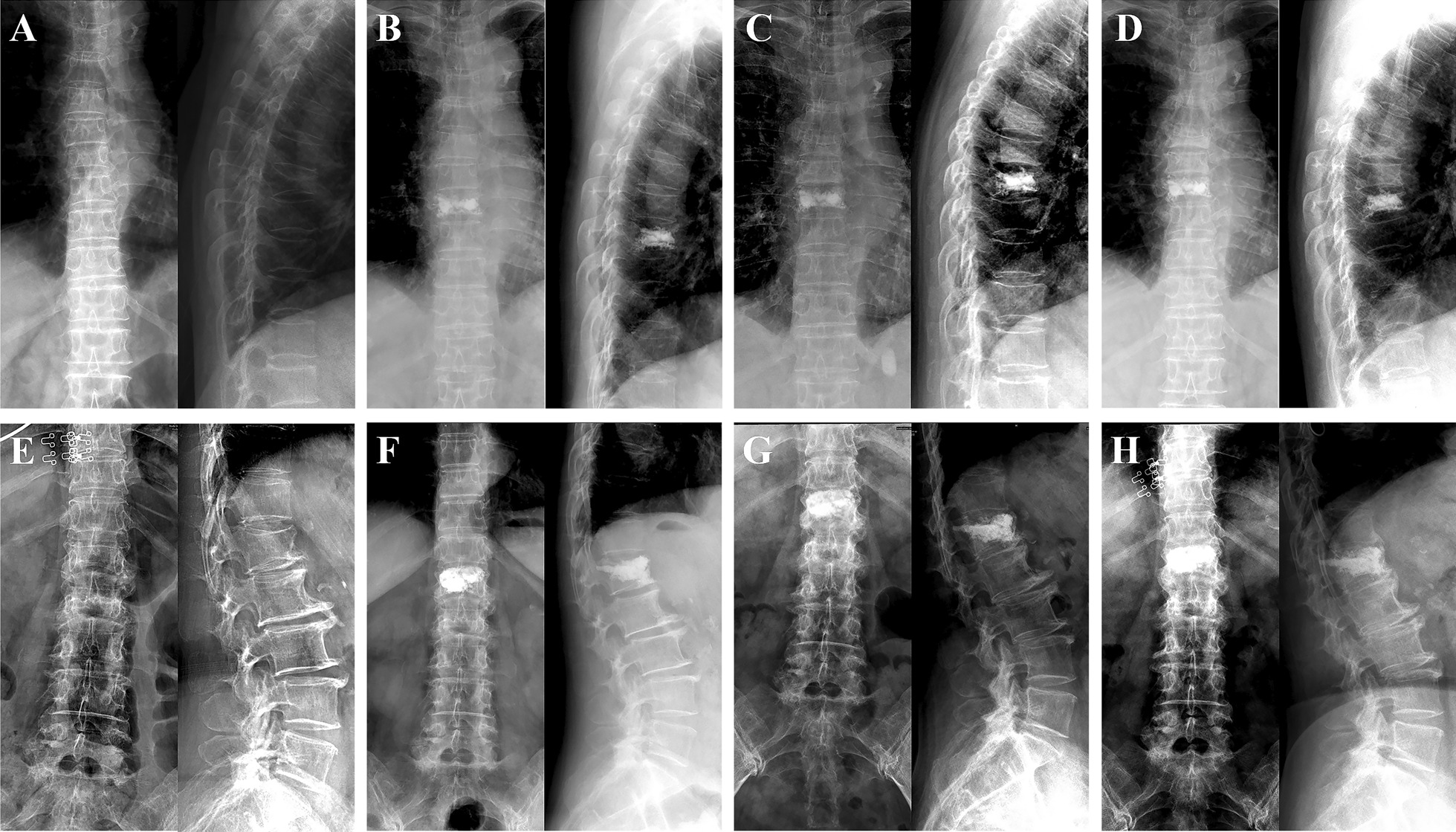
Anteroposterior and lateral vertebral body X-rays of typical cases in the T2DM group and control group. T8 fracture in the T2DM group’s typical case and L1 fracture in the control group’s typical case. **A**–**D** for the T2DM case. **E**–**H** for the control case. **A** and **E** Preoperation. **B** and **F** 2 days after operation. **C** and **G** 6 months after operation. **D** and **H** 12 months after operations. T2DM, type 2 diabetes mellitus

A total of 261 OVCF segments were included in this research, including 68 in the T2DM group and 193 in the control group. The anteroposterior and lateral film of the typical cases of the two groups before surgery, 2 days after surgery, 6 months after surgery and 12 months after surgery are shown in Fig. 5 (T8 fracture in the T2DM group’s typical case and L1 fracture in the control group’s typical case). The image measurement results of the two evaluators were generally reliable, with an intraclass correlation coefficient between 0.86 and 0.99 (95% confidence interval, *p* < 0.001) for the height of each portion of the vertebral body and an intraclass correlation coefficient between 0.87 and 0.96 (95% confidence interval, *p* < 0.001) for LCA.

Paired *t*-test showed that the APH, MPH, PPH and LCA of the fractured vertebral bodies in the T2DM group and the control group were significantly improved on the second day, the sixth month, and the twelfth month after surgery compared with those before surgery (*P* < 0.001). As shown in Table 3, the independent *t*-test revealed that there was no statistically significant difference in the percentage of height loss and LCA of each vertebral body between the T2DM group and the control group before PVP. The anterior portion and the middle portion in the T2DM group indicated a trend of slightly more compression than that of the control group, which were 27.45 ± 15.37% and 36.98 ± 13.02%, respectively. And they were 26.01 ± 14.62% and 35.80 ± 15.70% in the control group, both lower than those of the T2DM group. The LCA of 11.84 ± 7.06° in the T2DM group was greater than that of the control group, 10.25 ± 6.25°. Correspondingly, on the second day after operation, the correction of vertebral height produced by vertebroplasty in the T2DM group was also slightly greater than that in the control group. The APH recoveries were 19.49 ± 13.51% and 16.25 ± 13.72% in the respective group, and the MPH recovery were 30.71 ± 15.30% and 28.63 ± 16.80%, respectively. But there was no significant difference in the height recovery and LCA between the two groups. At 6 months after PVP, however, the vertebral LCA of T2DM patients was significantly larger than that of control patients (8.29 ± 4.90° vs 6.05 ± 5.18°, *P* = 0.002). The *P* values of the APH, MPH and PPH contrasts were all greater than 0.05. At 12 months after surgery, the two groups showed significant differences in all radiological measurements except PPH. The APH in the T2DM group restored 8.13 ± 12.90%, which was less than the recovery amount of 12.51 ± 14.92% in the control group (*P* = 0.032). The height recovery of the MPH in the T2DM group (19.07 ± 16.47%) was less than that in the control group (24.63 ± 17.67%, *P* = 0.024). And the LCA in the T2DM group was 8.87 ± 4.71°, greater than that in the control group (6.18 ± 5.04°, *P* < 0.001).

**Table 3:** Height and LCA of vertebral bodies in the T2DM group and control group before surgery, 2 days, 6 months and 12 months after surgery

	T2DM	Control	*P* value
*Height loss-before (%)*			
APH	27.45 ± 15.37	26.01 ± 14.62	0.493
MPH	36.98 ± 13.02	35.80 ± 15.70	0.579
PPH	9.51 ± 8.70	10.19 ± 10.03	0.621
LCA-before (°)	11.84 ± 7.06	10.25 ± 6.25	0.082
*Height restoration-2 days (%)*			
APH	19.49 ± 13.51	16.25 ± 13.72	0.094
MPH	30.71 ± 15.30	28.63 ± 16.80	0.369
PPH	7.06 ± 11.90	8.03 ± 11.97	0.566
LCA-2 days (°)	6.32 ± 4.36	6.15 ± 4.78	0.783
*Height restoration-6 months (%)*			
APH	11.35 ± 12.39	12.99 ± 14.54	0.408
MPH	21.69 ± 16.03	24.84 ± 17.62	0.197
PPH	5.03 ± 12.00	5.73 ± 12.45	0.689
LCA-6 months (°)	8.29 ± 4.90	6.05 ± 5.18	0.002*
*Height restoration-12 months (%)*			
APH	8.13 ± 12.90	12.51 ± 14.92	0.032*
MPH	19.07 ± 16.47	24.63 ± 17.67	0.024*
PPH	4.09 ± 12.32	5.29 ± 12.76	0.502
LCA-12 months (°)	8.87 ± 4.71	6.18 ± 5.04	< 0.001*

*APH*, anterior portion height; *LCA*, local Cobb angle; *MPH*, middle portion height; *PPH*, posterior portion height; *T2DM*, type 2 diabetes mellitus

*P < 0.05

### Complications

No nerve root injury, spinal cord injury, allergy, spinal infection or pulmonary embolism occurred during the entire follow-up. 3 cases of bone cement leakage occurred in the T2DM group, involving 3 vertebral bodies as T12, L1, and L1. 2 cases were type-C leakage, which leaked along the cortical defect to the upper intervertebral disc, and 1 case belonged to type-S leaking around the vertebral body. In the control group, there were 5 patients with bone cement leakage, involving 5 vertebral bodies: T7, T12, T12, L1 and L3. Among them, 3 cases were type-C leakage, and 2 cases were type-B leakage with the PMMA leaking along the vertebral basilar vein to the posterior border. Fisher's exact test demonstrated that there was no significant difference in the incidence of bone cement leakage between the two groups (Table 4, *P* = 0.438). There were 3 cases of new vertebral fractures other than the primary OVCF segment in the T2DM group, all of which were adjacent segment fractures, accounting for 5.3%. The shortest interval between the initial and the secondary was 3 months, and the longest interval was 13 months. There were 12 cases of new vertebral fractures in the control group, accounting for 7.5%, consisting of 6 cases of adjacent vertebrae and 6 cases of non-adjacent vertebrae fractures. The shortest interval was 2 months, and the longest interval was 47 months. All cases of new vertebral fracture in both groups were single-segment fractures, and there was no significant difference between the groups (*P* = 0.764).

**Table 4:** Complications of the T2DM group and control group

	T2DM (*N* = 57)	Control (*N* = 159)	*P* value
Bone cement leakage (*N*)	3 (5.3%)	5 (3.1%)	0.438
New OVCF (*N*)	3 (5.3%)	12 (7.5%)	0.764

*OVCF*, osteoporotic vertebral compression fracture; *T2DM*, type 2 diabetes mellitus

### The prognostic effect of OF classification on vertebral body height and Cobb angle

According to the preoperative fractures’ morphology of OF classification in Fig. 1, among all 261 OVCF vertebral segments, there were 96 (36.8%) OF 2 vertebral bodies, 90 (34.5%) OF 3 vertebral bodies, and 75 (28.7%) OF 4 vertebral bodies. There were no OF grades 1 and 5 fractures.

One-way ANOVA in Table 5 indicated that three different OF grades had statistically significant differences in the APH and MPH recovery and LCA of the vertebral body at 2 days, 6 months, and 12 months after PVP (except for *P* = 0.001 for MPH at 6 months, and 12 months postoperatively, *P* < 0.001 for the rest). However, there was no significant difference in the percentage of PPH recovery of the vertebral body (*P* > 0.05). In Table 6, the post-hoc analysis further verified the rehabilitative outcome of APH. OF grade 4 was better than OF grade 3, and OF grade 3 was better than OF grade 2, which was proved at all three time points after surgery (*P* < 0.05). The APH correction ratios of OF grade 2, 3, and 4 were 10.17 ± 11.17%, 16.91 ± 10.50%, and 26.18 ± 14.89% two days after the operation, respectively. The restoration effect of the MPH was different from that of APH. OF grade 4 was better than OF grade 2, and OF grade 2 was better than OF grade 3, which was also proved at the three postoperative time points (*P* < 0.05). The proportions were 27.22 ± 15.82%, 21.76 ± 12.65%, and 40.57 ± 15.18% for OF 2, 3 and 4 respectively. Compared with OF grade 4, OF grade 2 showed a significantly smaller LCA of the fractured vertebral body after surgery (*P* < 0.05). It also showed similar statistical results compared with OF grade 3 (*P* < 0.05). Nevertheless, the prognosis of LCA of OF grade 3 and 4 was not significantly different.

**Table 5:** One-way ANOVA for vertebral bodies height restoration and LCA of OF classification at 2 days, 6 months and 12 months after surgery

	OF 2	OF 3	OF 4	*P* value
*Height-2 days (%)*				
APH	10.17 ± 11.17	16.91 ± 10.50	26.18 ± 14.89	< 0.001*
MPH	27.22 ± 15.82	21.76 ± 12.65	40.57 ± 15.18	< 0.001*
PPH	8.94 ± 13.47	7.08 ± 10.46	7.14 ± 11.53	0.305
LCA-2 days (°)	3.65 ± 4.45	7.49 ± 3.49	7.89 ± 4.79	< 0.001*
*Height-6 months (%)*				
APH	6.92 ± 13.52	13.39 ± 11.37	18.80 ± 14.76	< 0.001*
MPH	23.23 ± 18.23	17.67 ± 13.39	32.63 ± 16.66	0.001*
PPH	6.53 ± 13.36	6.11 ± 11.09	3.62 ± 12.26	0.137
LCA-6 months (°)	3.46 ± 4.86	8.31 ± 4.15	8.67 ± 4.78	< 0.001*
*Height-12 months (%)*				
APH	5.65 ± 12.88	11.48 ± 12.81	18.55 ± 15.36	< 0.001*
MPH	22.60 ± 17.88	16.15 ± 13.46	32.35 ± 17.40	0.001*
PPH	6.28 ± 13.15	5.23 ± 11.53	3.01 ± 13.16	0.099
LCA-12 months (°)	3.78 ± 4.78	8.70 ± 4.00	8.66 ± 4.74	< 0.001*

*APH*, anterior portion height; *LCA*, local Cobb angle; *MPH*, middle portion height; *OF*, osteoporotic fracture; *PPH*, posterior portion height; *T2DM*, type 2 diabetes mellitus

*P < 0.05

**Table 6:** Post hoc analysis for vertebral bodies height restoration and LCA of OF classification at 2 days, 6 months and 12 months after surgery

	OF 2 versus OF 3	OF 2 versus OF 4	OF 3 versus OF 4
*Height-2 days*			
APH(%)	− 6.73 ± 3.50	− 16.0 ± 3.68	− 9.27 ± 3.73
*P* value	< 0.001*	< 0.001*	< 0.001*
MPH(%)	5.45 ± 4.22	− 13.3 ± 4.43	− 18.8 ± 4.49
*P* value	0.012*	< 0.001*	< 0.001*
PPH(%)	1.86 ± 3.45	1.80 ± 3.62	− 0.05 ± 3.67
*P* value	0.289	0.328	0.975
LCA-2 days (°)	− 3.83 ± 1.22	− 4.24 ± 1.29	− 0.40 ± 1.30
*P* value	< 0.001*	< 0.001*	0.545
*Height-6 months*			
APH(%)	− 6.46 ± 3.81	− 11.8 ± 4.00	− 5.40 ± 4.06
*P* value	0.001*	< 0.001*	0.009*
MPH(%)	5.56 ± 4.69	− 9.39 ± 4.92	− 14.9 ± 5.00
*P* value	0.02*	< 0.001*	< 0.001*
PPH(%)	0.41 ± 3.55	2.91 ± 3.73	2.49 ± 3.78
*P* value	0.816	0.126	0.197
LCA-6 months (°)	− 4.85 ± 1.33	− 5.21 ± 1.39	− 0.35 ± 1.41
*P* value	< 0.001*	< 0.001*	0.619
*Height-12 months*			
APH(%)	− 5.83 ± 3.93	− 12.9 ± 4.13	− 7.07 ± 4.19
*P* value	0.004*	< 0.001*	0.001*
MPH(%)	6.45 ± 4.72	− 9.75 ± 4.95	− 16.2 ± 5.03
*P* value	0.008*	< 0.001*	< 0.001*
PPH(%)	1.04 ± 3.64	3.26 ± 3.82	2.22 ± 3.88
*P* value	0.573	0.094	0.262
LCA-12 months (°)	− 4.92 ± 1.30	− 4.87 ± 1.37	0.05 ± 1.39
*P* value	< 0.001*	< 0.001*	0.948

*APH*, anterior portion height; *LCA*, local Cobb angle; *MPH*, middle portion height; *OF*, osteoporotic fracture; *PPH*, posterior portion height; *T2DM*, type 2 diabetes mellitus

*P < 0.05

### OF subgroup analysis of the influence of T2DM on the prognosis of vertebral body height and Cobb angle

As shown in Table 7, among the 68 OVCF vertebrae in the T2DM group, 21 (30.9%) were classified as OF grade 2, 23 (33.8%) were classified as OF grade 3, and 24 (35.3%) as OF grade 4. Among the 193 OVCF vertebrae in the control group, the number of vertebral bodies in OF grade 2, OF grade 3, and OF grade 4 were 75 (38.9%), 67 (34.7%), and 51 (26.4%), respectively. The proportion of OF grade 2 in the T2DM group was smaller than that of the control group, and the proportion of OF grade 4 was greater than that of the control group. But the rank sum test showed that there was no significant difference in the composition of OF grades between the two groups (*P* = 0.141).

**Table 7:** Number of OF classification of the T2DM group and the control group

	T2DM	Control	*P* value
Total (N)	68	193	0.141
OF 2	21 (30.9%)	75 (38.9%)	
OF 3	23 (33.8%)	67 (34.7%)	
OF 4	24 (35.3%)	51 (26.4%)	

*OF*, osteoporotic fracture; *T2DM*, type 2 diabetes mellitus

Comparing the T2DM group with the control group within the OF subgroup (Table 8), the OF grade 2 recovered less only in the percentage of MPH (14.82 ± 14.71% vs 24.78 ± 18.16%, *P* = 0.023) and LCA (5.65 ± 4.06 vs 3.26 ± 4.86°, *P* = 0.043) at 12 months postoperatively during the follow-up period. OF grade 3 not only recovered less in the anterior and middle parts at 12 months after surgery (APH: 5.40 ± 11.02% vs 13.57 ± 12.79%,* P* = 0.008; MPH: 11.22 ± 15.53% vs 17.84 ± 12.36%, *P* = 0.041), but there was a significant difference of T2DM patients in LCA at 6 months after operation (10.26 ± 3.59 vs 7.64 ± 4.15°, *P* = 0.008) and 12 months after operation (10.85 ± 3.79 vs 7.97 ± 3.83°, *P* = 0.002), compared with the control group. However, there was no statistically significant difference in the radiographic outcome of the OF grade 4 vertebrae between the T2DM group and the control group.

**Table 8:** Height restoration and LCA of OF classification at 2 days, 6 months, and 12 months after surgery in the T2DM group and control group

	T2DM	Control	*P* value
*OF 2*			
Height-2 days (%)			
APH	12.28 ± 12.47	9.58 ± 10.80	0.329
MPH	26.14 ± 15.36	27.52 ± 16.04	0.727
PPH	11.32 ± 16.82	8.28 ± 12.42	0.363
LCA-2 days (°)	3.70 ± 2.84	3.63 ± 4.82	0.952
Height-6 months (%)			
APH	6.26 ± 9.73	7.11 ± 14.45	0.801
MPH	17.55 ± 15.69	24.82 ± 18.66	0.106
PPH	8.32 ± 16.15	6.03 ± 12.56	0.490
LCA-6 months (°)	4.73 ± 4.33	3.10 ± 4.97	0.177
Height-12 months (%)			
APH	2.58 ± 9.71	6.51 ± 13.57	0.219
MPH	14.82 ± 14.71	24.78 ± 18.16	0.023*
PPH	8.01 ± 15.59	5.79 ± 12.45	0.497
LCA-12 months (°)	5.65 ± 4.06	3.26 ± 4.86	0.043*
*OF 3*			
Height-2 days (%)			
APH	17.55 ± 8.42	16.69 ± 11.18	0.736
MPH	24.17 ± 13.51	20.94 ± 12.34	0.294
PPH	6.54 ± 8.44	7.27 ± 11.13	0.775
LCA-2 days (°)	7.99 ± 4.18	7.31 ± 3.24	0.421
Height-6 months (%)			
APH	9.69 ± 10.38	14.66 ± 11.49	0.071
MPH	14.67 ± 15.74	18.70 ± 12.45	0.215
PPH	5.20 ± 10.83	6.42 ± 11.25	0.651
LCA-6 months (°)	10.26 ± 3.59	7.64 ± 4.15	0.008*
Height-12 months (%)			
APH	5.40 ± 11.02	13.57 ± 12.79	0.008*
MPH	11.22 ± 15.53	17.84 ± 12.36	0.041*
PPH	3.82 ± 11.37	5.72 ± 11.63	0.499
LCA-12 months (°)	10.85 ± 3.79	7.97 ± 3.83	0.002*
*OF 4*			
Height-2 days (%)			
APH	27.66 ± 14.41	25.49 ± 15.20	0.559
MPH	40.99 ± 11.41	40.37 ± 16.76	0.871
PPH	3.85 ± 8.39	8.69 ± 12.52	0.090
LCA-2 days (°)	7.05 ± 4.69	8.31 ± 4.83	0.276
Height-6 months (%)			
APH	17.40 ± 14.04	19.46 ± 15.18	0.577
MPH	32.05 ± 10.96	32.91 ± 18.85	0.836
PPH	2.01 ± 7.82	4.39 ± 13.87	0.434
LCA-6 months (°)	9.52 ± 4.93	8.27 ± 4.71	0.297
Height-12 months (%)			
APH	15.61 ± 13.88	19.94 ± 15.96	0.258
MPH	30.31 ± 12.68	33.31 ± 19.26	0.490
PPH	0.92 ± 9.09	3.99 ± 14.66	0.349
LCA-12 months (°)	9.80 ± 4.69	8.12 ± 4.70	0.152

*APH*, anterior portion height; *LCA*, local Cobb angle; *MPH*, middle portion height; *OF*, osteoporotic fracture; *PPH*, posterior portion height; *T2DM*, type 2 diabetes mellitus

*P < 0.05

## Discussion

This study retrospectively analyzed 216 OVCF patients in our hospital from January 1, 2016 to June 30, 2021, to compare the vertebral height restoration and local Cobb angle of compression fractures, subjective pain symptoms and incidence of complications after PVP in T2DM patients and controls. Additionally, subgroups according to the OF classification were analyzed. The results illustrated that PVP surgery can quickly and significantly relieve the symptoms of low back pain and help early functional recovery. Besides, it can effectively restore the height and angle of the lost part of the compressed vertebral body, and rehabilitate the biomechanical stability. Compared with the general population, the degree of compression of the fractured vertebra and the degree of recovery of the height and angle obtained immediately after surgery were not different for T2DM patients. However, the LCA was worse than that of the control since the sixth month after surgery. And the surgical benefit of the APH and MPH was worse from 12 months after PVP. There was no difference in the complications of PVP surgery between the two groups. In terms of preoperative OF classification, OF 4 have a larger LCA after operation than OF 2, but there is no difference with OF 3. The postoperative rehabilitative outcome of the APH in OF 4 is better than OF 3, and OF 3 is better than OF 2. As for MPH, OF 4 is better than OF 2, and OF 2 is better than OF 3. The subgroup analysis of OF classification between T2DM patients and the control showed that there was no difference in the quantity distribution of OVCF types at all levels of OF. In respect of MPH and LCA, it’s worth noting that the morphology of OF 2 vertebral bodies in T2DM patients was worse than that of the general population at 12 months postoperatively. In OF 3 vertebral bodies, T2DM patients were worse than the general population in LCA at 6 months after surgery, and in the degree of APH and MPH recovery and LCA at 12 months after surgery. However, there was no difference in the radiological outcomes of OF 4 between the two populations.

This article confirmed the rapid and significant curative effect of PVP in the measurement of the height and angle of the lateral spine X-ray on the second postoperative day in Table 3. Furthermore, a total of 12-month follow-up period reflected the good mid-term maintenance efficacy of the PVP on the vertebral morphology. The further plan for the research is to collect the imaging data for the long-term maintenance effect of 5 years after surgery. The included population all received unilateral PVP because of its less trauma, shorter operation time, lower probability of nerve injury complications, lower radiational exposure of patients and medical staff, and lower leakage rate of bone cement compared with bilateral PVP. Moreover, a previous meta-analysis with 876 patients suggested that the unilateral approach bringing about pain alleviation and vertebral height and LCA correction, had no significant difference from bilateral puncture, but with a lower risk of PMMA leakage [14].

The bone damage in T2DM originates from many mechanisms. The basic research of Cassidy et al. and Rahman et al. discovered that both type 1 and 2 DM were capable of inhibiting the proliferation and differentiation of stem cells and increase fat accumulation to inhibit bone remodeling, thereby reducing bone intensity and giving rise to the impairment in density and microstructure [15, 16]. A clinical investigation with 5169 patients suggested that whether because of insulin insufficiency or resistance, immoderately high blood glucose levels can reduce osteocalcin and impair the bone synthesis capacity of osteoblasts, thereby reducing BMD and significantly increasing the risk of osteoporotic fracture [17]. Apart from that, insulin resistance in such patients can lead to the decreased sensitivity of stem cell surface receptors to cytokines and the inhibition of the binding of osteoblast surface receptors to insulin, which in turn reduces the production of alkaline phosphatase from osteoblasts, which also impairs its activity causing lower bone intensity [18]. Under the continuous influence of high glucose and high advanced glycation end products microenvironment, the separation degree of trabecular bone is significantly increased and the structure gets hypertrophic, which further affects the bone strength. It may be the reason why the morphological prognosis of spinal cancellous bone gradually lags behind the general population [19]. Since bone microvessels are vital supply channels for nutrition, research on vascular holes in bone tissue can indicate the nutritional status of bone, the susceptibility to fractures and the healing ability to fractures, the latter being more important for postoperative patients. Microangiopathy in T2DM is usually not only manifested in retina and kidney, but also degenerates blood vessels in bone tissue, with its cortical bone volume thickness damaged and porosity increased more significantly [20]. However, there is yet no conclusion whether it is due to the degeneration of the blood vessels from diabetes itself damaging the bone tissue, or the bone degeneration caused by OP first causes the reduction of microvascular cavities, further leading to the impairment of the prognosis. But the synergistic relationship between the T2DM and the occurrence and prognosis of OVCF as mentioned above was confirmed [21]. Compared with the control group, the LCA of the fractured vertebrae of the T2DM patients began to escalate since the sixth month after PVP, and the recovery of APH and MPH was worse since the twelfth month after PVP. The APH, MPH and LCA of the vertebral body in this group showed a trend of continuous damage during the prognostic follow-up period. This conclusion could be mutually confirmed by studies mentioned above, suggesting that T2DM still has damage to the vertebral cortex and cancellous microstructure in patients after surgery. High-resolution peripheral quantitative computed tomography in a mouse model of T2DM indicated the following results: trabecular bone increased in thickness but decreased in amount, in consequence of increased trabecular bone separation, reduced volume fraction, and decreased mechanical stability [21]. Clinically, although the trabecular bone in the T2DM group was thicker in structure, the amount was significantly reduced than that in the control group of the same age [22]. Partially different from it, after adjusting for BMI, age, hypertension, and chronic kidney disease, Farr showed no significant difference in BMD and trabecular microstructure between T2DM patients and controls, but decreased cortical bone thickness, increased porosity, and decreased bone intensity. The mean HbA1c level in the past 10 years was negatively correlated with bone intensity [23]. The conclusions of these previous clinical studies may explain why the vertebral body still has a minor degree of compression deformation in the anterior and middle parts after PVP.

First published by the German Society for Orthopedics and Trauma in 2018, the OF classification has solid interobserver reliability with *κ* = 0.63. Compared with the previous Genant semi-quantitative grading system and the Sugita prognostic classification system, it is easier to utilize and easier to distinguish the fractured shape in clinic, and has given treatment recommendations for each grade [9], whereas its prognostic value is unclear. Palmowski's immediate postoperative imaging analysis confirmed that the subgroup of patients with higher preoperative OF grade showed higher postoperative benefits in terms of radiological parameters after vertebroplasty [24]. It also proved that OF classification is an appropriate tool for sorting vertebral fractures and is a crucial factor to consider when deciding whether to treat conservatively or surgically [24]. Compared with the report, this subject extended the follow-up period to 12 months, and the study of OF classification reached similar but not completely equivalent conclusions. It was found that the PVP benefit for APH was OF 4 > OF 3 > OF 2, while the benefit of the MPH was OF 4 > OF 2 > OF 3. The LCA of OF 2 was always smaller than that of OF 3 and 4 from preoperative to 12 months after operation, while the LCA of OF 3 and OF 4 did not differ postoperatively. This conclusion is highly correlated with the preoperative morphology and mechanical stability when grading. In the subgroup analysis of T2DM patients, the recovery of the MPH of OF 2 was worse than that of the general population from 12 months after operation, and the recovery of both the APH and MPH of OF 3 got worse from 12 months after operation. It suggested that, for these two grades, T2DM has a negative influence on the prognostic repair of the fractured area. However, in OF 4, no prognostic differences in vertebral morphology and clinical symptoms were found between T2DM patients and the general population, probably due to the fact that the degree of compressive injury was more severe than that of OF 2 and 3, and the 1-year follow-up could merely distinguish the benefit of PVP in two groups. To date, there was no study reporting the prognostic significance of OF classification in short- or long-term. And in response to these results, we plan to include the vertebral body X-ray morphology and clinical symptoms of OVCF patients in the fifth year after PVP, in order to obtain longer-term and more accurate results of the prognostic impact of T2DM on such patients.

Complications after PVP include nerve root injury, bone cement leakage, bone cement allergy, recurrent vertebral fracture, spinal infection, pulmonary embolism, etc. Only bone cement leakage and new vertebral fracture were observed in the enrolled population. Ren's investigation demonstrated that bone cement viscosity, infusion volume, vertebral body wall injury, and history of lung disease were risk factors for the cement leakage [25]. Although the proportion of PMMA leakage in the T2DM group was higher than that in the control group (5.3% vs 3.1%), there was no significant difference between the groups. Besides, no study has suggested that T2DM was a risk factor for cement leakage after PVP. In Chen’s retrospective study, he reached similar conclusions to the previous scholars on the risk factors of bone cement leakage, and illustrated that diabetes and the change of LCA after PKP is positively correlated with the postoperative adjacent vertebral fractures [8]. The recurrence rate of OVCF in this research was relatively low, with 5.3% in the T2DM group, and there was no difference between the groups. It may be because the routine recommendation for each patient by our doctors to have a balanced diet, regularly monitor and control blood glucose, take DXA examination once a year, receive daily oral calcium and vitamin D supplementation, and anti-OP therapy administering with zoledronic acid or teriparatide or denosumab according to the bone metabolism results and economic condition.

This study has certain limitations. Although there might not be obvious changes in vertebral body morphology in the long-term without violent trauma, the 12-month follow-up time of this prognostic study still needs an extension, and the long-term efficacy of PVP needs further research. Additionally, the data on bone metabolism, blood glucose, and HbA1c were incomplete during the postoperative follow-up period, resulting in unclear glycemic control during follow-up and an inability to compare with perioperative levels. Last but not least, the specific anti-OP and antidiabetic regimens of each patient are inconsistent according to their characteristics, which may affect the prognosis of vertebral morphology and clinical symptoms. Though the antidiabetic regimens varied between individuals, including biguanides, α-glycosidase inhibitors, sulfonylureas, dipeptidyl peptidases inhibitor, glucagon-like peptide-1 receptor agonist and insulin, our clinicians managed to recommend routine follow-up in endocrinology department to patients and recorded.

## Conclusion

PVP can effectively relieve the symptoms of low back pain in patients with OVCF, contributing to early functional recovery. The degree of fractured vertebral body compression and the recovery of the height and angle obtained immediately after surgery in T2DM patients were not different from those in the general population. However, the vertebral body morphology of T2DM patients was worse than the general population since the sixth month after surgery. OF classification has a good prognostic reference value in morphology. The shape of OF 2 and 3 in T2DM patients was worse than that of the general population from 12 and 6 months respectively after surgery, and the shape of OF 4 has no difference from the general population within 12 months after surgery. Orthopedic surgeons should instruct T2DM patients to routinely monitor and control blood glucose, and give standardized individualized anti-osteoporosis regimens.

## Supplementary Information

**Additional file 1**: Title of data: Fractured segments. Description of data: Fractured segments of every included de-identified participant in the T2DM and control group.

## Data Availability

De-identified laboratory and radiological data of all the included patients can be available upon reasonable request from qualified academic investigators by contacting the e-mail of the corresponding author and stating the purpose of reuse.

## Abbreviations

APH: Anterior portion height
BMD: Bone mineral density
BMI: Body mass index
HbA1c: Glycosylated hemoglobin
LCA: Local Cobb angle
MPH: Middle portion height
ODI: Oswestry disability index
OF: Osteoporotic fracture
OP: Osteoporosis
OVCF: Osteoporotic vertebral compression fracture
PMMA: Polymethyl methacrylate
PPH: Posterior portion height
PVP: Percutaneous vertebroplasty
T2DM: Type 2 diabetes mellitus
VAS: Visual analogue scale
